# MRI-derived IVIM-modelled diffusion-weighted imaging does not predict clinical outcomes in fontan-associated liver disease: to monitor or not to monitor the liver?

**DOI:** 10.3389/fped.2026.1816094

**Published:** 2026-06-22

**Authors:** Gaston van Hassel, Frans J. C. Cuperus, Paul E. Sijens, Tineke P. Willems, Eryn T. Liem, Elke S. Hoendermis, Joost P. van Melle, Rolf M. F. Berger

**Affiliations:** 1 Center for Congenital Heart Diseases, Paediatric Cardiology, Beatrix Children’s Hospital, University of Groningen, University Medical Centre Groningen, Groningen, Netherlands; 2 Department of Gastroenterology and Hepatology, University of Groningen, University Medical Center Groningen, Groningen, Netherlands; 3 Department of Radiology, University of Groningen, University Medical Center Groningen, Groningen, Netherlands; 4 Department of Cardiology, University of Groningen, Center for Congenital Heart Diseases, University Medical Center Groningen, Groningen, Netherlands

**Keywords:** Fontan, liver, magnetic resonance imaging (MRI), outcome, risk factors

## Abstract

**Background:**

Fontan-associated liver disease (FALD) encompasses the spectrum of hepatic alterations in patients after Fontan palliation. FALD can progress from congestive hepatopathy to fibrosis to cirrhosis with portal hypertension, and is associated with an increased risk of hepatocellular carcinoma (HCC). Current guidelines recommend intensive yearly folIow-up to assess FALD severity. However, the opted surveillance methods are unable to distinguish hepatic congestion from fibrosis or cirrhosis. Additionally, current literature provides limited evidence that FALD surveillance prevents adverse events. Intravoxel incoherent motion (IVIM) modelled diffusion-weighted imaging (DWI) allows for non-invasive differentiation between hepatic congestion and fibrosis, and has been suggested as a surveillance strategy in FALD. The objective of this study is to investigate the prognostic value of MRI-derived IVIM-modelled DWI in patients with a Fontan circulation.

**Methods:**

In a prospective study, 59 consecutive Fontan patients underwent hepatic MRI for IVIM-modelled DWI measurements. Apparent diffusion coefficient (ADC) and parameters of hepatic congestion—namely, the fast diffusion component (D_fast_, reflecting microperfusion-related water movement), the fraction of microperfusion (f_fast_, indicating the proportion of signal from microvascular perfusion), and fibrosis (slow diffusion component (D_slow_), representing restricted water diffusion in dense tissue)—were measured. The association between DWI measurements and a composite endpoint of all-cause mortality and Fontan-related hospitalization was assessed using Cox-regression analyses.

**Results:**

Mean cohort age was 19 ± 8 years, 51% female. Mean ADC was (0.82 ± 0.11) × 10^−3^ mm^2^/s, D_fast_ (20.74 ± 6.60) × 10^−3^ mm^2^/s, f_fast_ 29 ± 05%, and D_slow_ (0.77 ± 0.15) × 10^−3^ mm^2^/s. During a median follow-up time of 9 (Q1-Q3: 5–11) years, the primary endpoint occurred in 33 patients (59%). We found no significant association between IVIM-modelled DWI parameters at inclusion and the composite primary endpoint at follow up (all *p* > 0.05).

**Conclusion:**

IVIM-modelled DWI addresses a key diagnostic challenge in FALD by enabling distinction between hepatic congestion and fibrosis. However, even after this distinction, we did not find a relationship between IVIM-modelled DWI and clinical outcomes. These findings, in combination with current literature data, which also fail to demonstrate a relation between other non-invasive FALD assessment tools and outcome, raise the question whether the recommended intensive FALD monitoring strategies are justified.

## Introduction

Since its initial description in 1971, the Fontan palliation has improved both quality of life and clinical outcomes in patients born with a functionally univentricular heart by alleviating volume overload for the single ventricle as well as cyanosis (1). Although the hemodynamic changes of the Fontan circulation are generally well tolerated in early childhood—following Fontan completion at a median age of 4 years—adolescents and young adults with a Fontan circulation experience high levels of morbidity and mortality (1, 2).

Fontan-associated liver disease (FALD) is one of the co-morbidities faced by patients with a Fontan circulation that has raised concerns. FALD is a spectrum of congestive hepatopathy, which progresses from hepatic congestion to fibrosis and may ultimately advance to cirrhosis with portal hypertension. In addition, FALD is associated with an increased risk of hepatocellular carcinoma (HCC) (3, 4). FALD is assumed to arise from both sustained hepatic venous congestion and impaired hepatic perfusion (3). As systemic perfusion worsens and venous congestion increases over time, the severity of hepatic fibrosis progresses with the duration of the Fontan circulation (3, 5).

**Figure 1:**
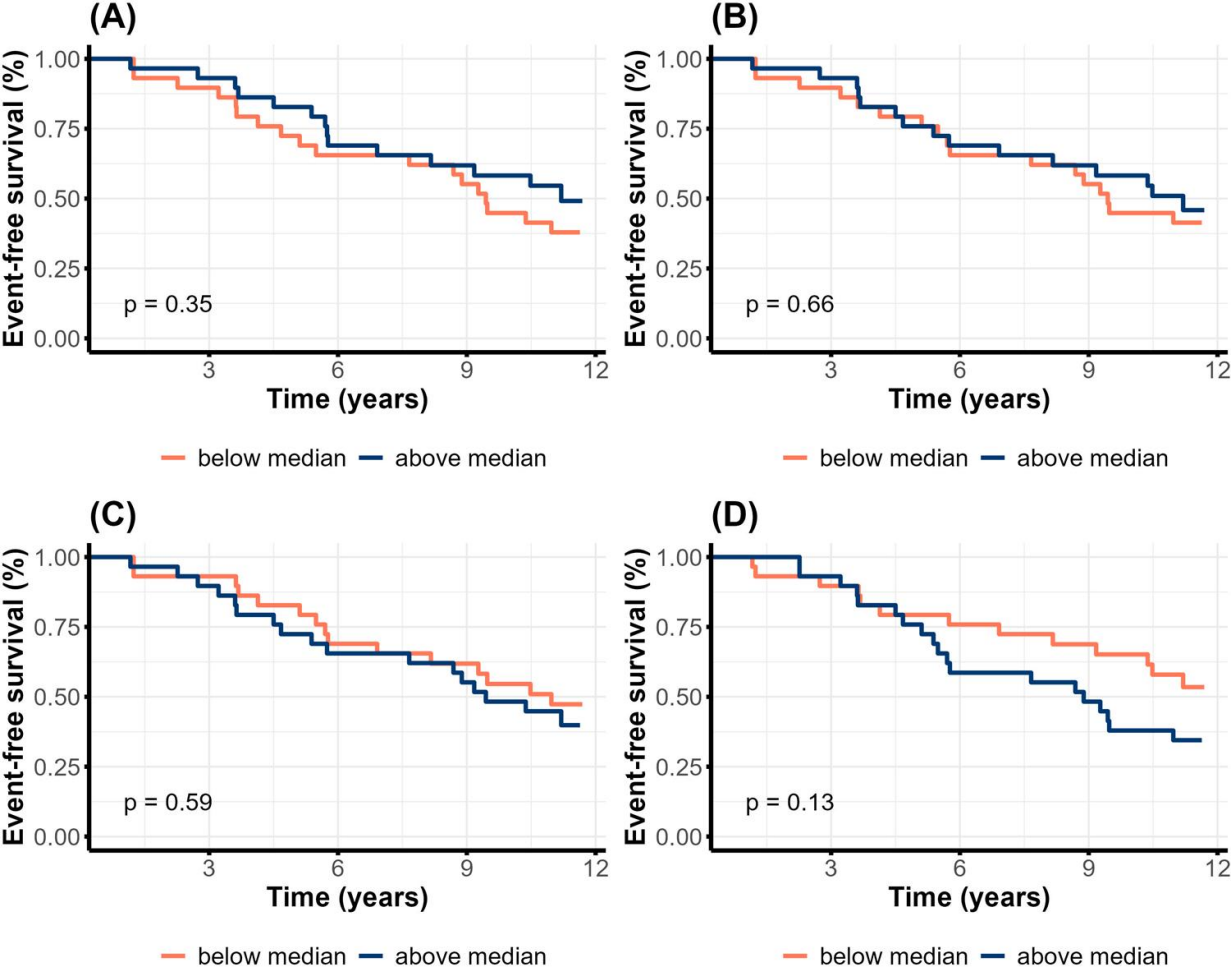
**(A)** kaplan–meier plot for the primary endpoint stratified by median ADC (0.84 × 10^−3^ mm^2^/s). **(B)** Kaplan–Meier plot for the primary endpoint stratified by the median of D_slow_ (0.79 × 10^−3^ mm^2^/s). **(C)** Kaplan–Meier plot for the primary endpoint stratified by the median of D_fast_ (19.37 × 10^−3^ mm^2^/s). **(D)** Kaplan–Meier plot for the primary endpoint stratified by the median of F_fast_ (28.7%). *P* value represents the evidence against the null hypothesis of no difference in survival across the stratifications, as calculated using log-rank test.

Current guidelines recommend an intensive, lifelong, FALD monitoring, including annual hepatic surveillance, consisting of laboratory testing, hepatic ultrasound and consultation with a hepatologist (6, 7). However, considerable debate remains regarding how to define FALD severity and what constitutes an optimal surveillance strategy, despite the release of a European consensus statement in 2023 (8, 9). Liver biopsy is regarded as the gold standard to evaluate the degree of hepatic fibrosis/cirrhosis, and thus of FALD progression (8). Conversely, due to its invasive nature and associated risk of complications, routine biopsy is unfavourable for surveillance protocols. Moreover, it has been recognized that the fibrotic changes may show a heterogeneous distribution across the various liver lobes (10). Therefore, several less invasive tools have been suggested to monitor FALD, including serum biomarkers such as platelet count, elastography (FibroScan), and imaging techniques using ultrasound, computer tomography (CT), or magnetic resonance imaging (MRI). Nevertheless, most of these recommended methods are limited in their inability to distinguish between hepatic congestion and other, more advanced stages of FALD (Figure 1) and thus lack specificity for grading or tracking FALD progression, especially in its early stages without portal hypertension (11, 12).

Previous studies have suggested magnetic resonance diffusion-weighted imaging (DWI), a form of MRI that measures the random Brownian motion of water molecules within a tissue voxel, as a quantitative imaging biomarker to assess the liver in patients with a Fontan circulation. These studies have demonstrated lower hepatic apparent diffusion coefficient (ADC) values in these patients compared to controls, suggesting congestive hepatopathy. These ADC-values correlated negatively with the time since Fontan completion (13–15). Furthermore, this technique has the potential to non-invasively distinguish hepatic congestion from hepatic fibrosis in patients with a Fontan circulation by intravoxel incoherent motion (IVIM) modelling (13–15). Based on these unique characteristics, IVIM-modelled DWI has been suggested as a non-invasive alternative for liver biopsy to monitor FALD progression. In the current study we aim to investigate the prognostic value of hepatic characteristics as obtained from MRI-DWI with outcome in patients with a Fontan circulation.

## Methods

### Study population, design, and data collection

For this single-centre study, we collected follow-up data of all 59 patients with a Fontan circulation ≥ 10 years of age who were included in the FAIR-study (**F**ontan circulation: **A**nalysis of its **I**mpact on quality of life and **R**ate of deterioration) between January 2012 and October 2013 at the University Medical Center Groningen (The Netherlands) (16, 17). In this institution, all patients with a Fontan circulation are included in a standardized follow-up protocol, and all patients and caregivers were asked for written informed consent for additional study investigations, including MRI of the liver, performed at the same time as regular follow-up investigations.

At baseline of the FAIR-study, all included patients underwent standardized testing, including cardiopulmonary exercise test, magnetic resonance imaging (MRI) of heart and liver, echocardiography, and venipuncture for laboratory investigations. Cardiac function was evaluated by MRI. The imaging protocol and its analyses were performed as previously described (16). MRI-measurements included end-diastolic volume indexed by BSA (combined hypoplastic and systemic ventricle volumes), ejection fraction, and cardiac index. Blood samples were collected on the same day as the MRI-scan for most patients, and for the remaining patients within one week. Demographic, clinical, and cardiac-related characteristics at baseline were extracted from patient records.

The institutional ethics committee approved the study (METc 2012080). This study was conducted in accordance with the principles outlined in the Declaration of Helsinki.

### Patient and public involvement

No patients nor the public were involved in the design of this study.

### MRI protocol and diffusion-weighted imaging analysis

During cardiac MRI at baseline, diffusion-weighted imaging (DWI) (the measurement of random microscopic motion of water molecules in tissue) of the liver was acquired using a commercially available 1.5T scanner (Magnetom Aera, Siemens Medical Solutions, Erlangen, Germany).The protocol has been described previously (13, 17). Briefly, nine series of DWI (b-values: 0–1,750 s/mm^2^) were acquired using a spin-echo based echo-planar imaging sequence with prospective acquisition correction (PACE) respiratory triggering and spectral attenuated inversion recovery (SPAIR) fat suppression. PACE is a navigator-triggered respiratory motion correction technique that monitors diaphragmatic position and trigger data acquisition at regular moments in the respiratory cycle in order to reduce motion artifacts in free-breathing MRI sequences. SPAIR is a hybrid fat suppression technique to provide more homogeneous fat suppression in hepatic MRI. Regions-of-interest (ROIs) were drawn in 8 liver segments according to the Couinaud-Bismuth classification, avoiding major blood vessels.

Analysis was performed using monoexponential and biexponential fitting procedures based on the intravoxel incoherent motion (IVIM) model.

$$\frac{S}{S_{0}}=f_{\mathrm{fast}}\cdot \exp\;(-b\cdot D_{\mathrm{fast}})+\;f_{\mathrm{slow}}\cdot \exp\;(-b\cdot \;D_{\mathrm{slow}})$$

S_0_ refers to the maximum signal intensity at b-value = 0 s/mm^2^. D_fast_ represents the diffusion coefficient of the fast-decaying component in the IVIM model, reflecting hepatic microperfusion or microcapillary blood flow. F_fast_ is the fraction of MRI signal attributed to the fast-decaying perfusion component in the IVIM model, representing the proportion of voxel volume occupied by flowing blood in capillaries. D_slow_ represents the diffusion coefficient of the slow-decaying component in the IVIM model, reflecting true molecular diffusion of water in tissue with minimal perfusion contamination. The Nelder-Mead simplex direct search method with bound constraints was used for fitting. Apparent diffusion coefficient (ADC) was obtained using a mono-exponential fit of all b-values. ADC is a quantitative parameter that reflects the overall diffusion of water molecules in tissue.

### Outcome

For this study, follow-up data were collected up to January 1, 2024. All patients underwent regular and standardized tests in accordance with our centre's Fontan protocol, including physical examination, echocardiography, cardiac MRI, exercise tests and venipuncture. The primary (combined) endpoint was defined as Fontan-related hospitalization, listing for heart transplantation, and death. Fontan-related hospitalization was defined as hospitalization for heart-/circulatory failure needing intravenous diuretics, hemodynamic evaluation of failing Fontan, arrhythmia, thromboembolic events, protein losing enteropathy (PLE), ascites, or any cardiac-related intervention (i.e., percutaneous treatment of Fontan pathway obstructions, enlargement of ventricular septal defect, aortic valve/arch replacement and cardiac ablation). The secondary (also combined) endpoint, further referred to as hepatic endpoint, was defined as an overnight admission for FALD (defined as clinical or biochemical deterioration in liver function, or suspected HCC requiring an overnight admission for diagnostic biopsy), PLE, HCC, or ascites.

### Statistical analyses

Baseline data are as mean ± SD or median (Q1-Q3), depending on distribution and as number (percentage) for categorical variables. Normality was evaluated by visual inspection of histograms and Q-Q plots.

Longitudinal univariable Cox proportional hazards analyses were performed to investigate the association between ADC, D_slow_, D_fast_, F_fast_, treated as a continuous variable, and the primary as well as the secondary endpoint. The assumption of proportional hazards was not violated in any of the models, as assessed using Schoenfeld tests. Hazard Ratios (HR) of DWI are presented with 95% confidence intervals (95% CI). Additionally, Kaplan–Meier curves were fitted for the primary endpoint over time, stratified by high vs. low values for all four MRI-derived measurements.

All analyses were performed, and all figures were designed using the R statistical software (version 4.3.2). A 2-sided *P* < 0.05 was considered to indicate statistical significance.

## Results

### Baseline characteristics

Baseline characteristics of the 59 patients who were included in the original FAIR study have been reported previously (17). An overview can be found in Table 1. The mean age at inclusion in the FAIR study was 19.1 ± 7.5 years, and 51% was female. Of the included patients, 32 were children (54%) and 27 were adults (46%). The most frequent underlying cardiac diagnosis was tricuspid atresia (37%), followed by double inlet left ventricle (17%). No patients had heterotaxy. The majority of the population was palliated with a lateral tunnel or an extracardiac conduit (48% and 37%, respectively). Median time since completion of the Fontan circulation was 11 (Q1–Q3: 7–18) years. At inclusion, 56%, 39% and 5% of the patients were classified as New York Heart Association (NYHA) functional class I, II, and III, respectively.

**Table 1:** Patient characteristics at inclusion of the FAIR-study (17).

Characteristics	*N* = 59
Age, years	19.1 ± 7.5
Female, *n* (%)	30 (51%)
Underlying diagnosis, *n* (%)	
Tricuspid atresia	22 (37%)
DILV	10 (17%)
PA with intact ventricular septum	9 (15%)
Unbalanced VSD/AVSD	9 (15%)
Heterogeneous anomalies	6 (10%)
HLHS	3 (5%)
Type of Fontan circulation, *n* (%)	
Atriopulmonary connection	7 (12%)
TCPC lateral tunnel	28 (48%)
TCPC extracardiac conduit	22 (37%)
Kawasima	2 (3%)
NYHA class, *n* (%)	
I	33 (56%)
II	23 (39%)
III	3 (5%)
Cardiac function	
Ejection fraction, (%)	56 ± 8
Cardiac index, L/min/m^2^	3.1 ± 0.9
End-diastolic volume index, mL/m^2^	78 ± 21
Laboratory measurements	
AST, U/L	32 ± 10
ALT, U/L	28 ± 9
AST/ALT ratio	1.2 ± 0.4
*γ*GT, U/L	73 ± 60
Alkaline Phosphatase, U/L	107 (78–224)
Total bilirubin, µmol/L	14 (10–17)
Albumin, g/L	48 (46–50)
MELD-XI	9.4 (9.4–9.4)
Fib-4	0.5 (0.4–0.7)
DWI measurements	
ADC, (×10^−3^ mm^2^/s)	0.82 ± 0.11
D_slow_, (×10^−3^ mm^2^/s)	0.77 ± 0.15
D_fast_, (×10^−3^ mm^2^/s)	20.74 ± 6.60
F_fast_, %	29 ± 05

*DILV*, double inlet left ventricle; *PA*, pulmonary valve atresia; *VSD/AVSD*, ventricular septum defect/atrioventricular septum defect; *HLHS*, hypoplastic left heart syndrome; *TCPC*, total cavopulmonary connection; *NYHA*, New York Health Association; *AST*, aspartate transaminase; *ALT*, alanine transaminase; *γGT*, gamma-glutamyl transferase; *ADC* apparent diffusion coefficient; *D_slow_* true diffusion coefficient; *D_fast_* pseudo diffusion coefficient; *F_fast_* percentage of diffusion attributed to perfusion.

At baseline, mean aspartate transaminase (AST) was 32 ± 10 U/L, alanine transaminase (ALT) was 28 ± 9 U/L, glutamyl transferase (*γ*-GT) was 73 ± 60 U/L, and AST/ALT ratio was 1.2 ± 0.4. Median model for end-stage liver disease excluding internationalized-normalized-ratio (MELD-XI) score was 9.4 (9.4–9.4) and median fibrosis-4 (Fib-4) score was 0.5 (0.4–0.7).

Mean MRI-derived indexed end-diastolic volume was 78 ± 21 mL/m^2^, ejection fraction was 56 ± 8%, and cardiac index was 3.1 ± 0.9 L/min/m^2^.

Liver IVIM-modelled DWI showed a mean ADC values for the eight regions of the liver of 0.82 × 10^−3^ ± 0.11 × 10^−3^ mm^2^/s, a mean D_fast_ of (20.74 ± 6.60) × 10^−3^ mm^2^/s, f_fast_ of 29 ± 5%, and a mean D_slow_ of (0.77 ± 0.15) × 10^−3^ mm^2^/s.

### Longitudinal associations of DWI parameters with outcome

During a median follow up period of 9 years (Q1-Q3: 3–11) since MRI examination the primary endpoint occurred in 33 patients (59%) and included 2 deaths, 4 hospitalizations due to either HCC (*n* = 2), PLE (*n* = 1) or ascites (*n* = 1), and 27 Fontan-related hospitalizations due to either Fontan failure (*n* = 3), arrhythmia (*n* = 12), thrombo-embolic events (*n* = 2), cardiovascular interventions (*n* = 9), or haemoptysis (*n* = 1).

Cox regression analysis on a continuous scale did not show a statistically significant association between ADC and the primary endpoint (HR 0.74; 95% CI 0.04–14.82; *p* = 0.8), D_slow_ (HR 0.43; 95% CI 0.05–3.92; *p* = 0.5), D_fast_ (HR 1.00; 95% CI 0.99–1.06; *p* = 0.9), and F_fast_ (HR 1.01; 95% CI = 0.99–1.01; *p* = 0.06) (Table 2). Similarly, stratification of these parameters into high vs. low did not show an association with the primary endpoint (Figure 1).

**Table 2:** Univariable Cox regression analysis for the association between IVIM-modelled DWI and the primary and secondary endpoints.

Measurement	HR	95% CI	*P* value
Primary endpoint			
ADC, (×10^−3^ mm^2^/s)	0.74	0.04 to 14.82	0.8
D_slow_, (×10^−3^ mm^2^/s)	0.43	0.05 to 3.92	0.5
D_fast_, (×10^−3^ mm^2^/s)	1.00	0.99 to 1.06	0.9
f_fast,_	1.01	0.99 to 1.01	0.06
Secondary endpoint			
ADC, (×10^−3^ mm^2^/s)	0.01	0.00 to 4.31	0.1
D_slow_, (×10^−3^ mm^2^/s)	0.06	0.00 to 9.46	0.3
D_fast_, (×10^−3^ mm^2^/s)	1.07	0.96 to 1.20	0.2
f_fast,_	1.00	0.99 to 1.02	0.5

The primary endpoint is a combined endpoint defined as Fontan-related hospitalization (i.e., hospitalization for heart-/circulatory failure needing intravenous diuretics, hemodynamic evaluation of failing Fontan, arrhythmia, thromboembolic events, protein losing enteropathy (PLE), ascites, or any cardiac-related intervention), listing for heart transplantation, and death.

Secondary endpoint is a combined endpoint defined as an overnight admission for FALD, PLE or ascites.

*ADC*, apparent diffusion coefficient; *D_slow_*, true diffusion coefficient; *D_fast_*, pseudo diffusion coefficient; f*_fast_*, fraction of diffusion attributed to perfusion; *HR*, hazards ratio; *95% CI*, 95% confidence interval.

In addition, ADC values at baseline did not predict the occurrence of the secondary endpoint (i.e., overnight admission for FALD, PLE, HCC, or ascites) during follow up (HR 0.01; 95% CI 0.00–4.31; *p* = 0.1), nor did D_slow_ (HR 0.06; 95% CI 0.00–9.46; *p* = 0.3), D_fast_ (HR 1.07; 95% CI 0.96–1.2; *p* = 0.2), or f_fast_ (HR 1.00; 95% CI = 0.99–1.02; *p* = 0.5) (Table 2).

Mann–Whitney *U*-test were performed to analyse the difference in IVIM-modelled DWI parameters between patients with and without a secondary endpoint. There were no differences in ADC (0.83 ± 0.11 vs. 0.73 ± 0.10 × 10^−3^ mm^2^/s; *p* = 0.9), D_slow_ (0.78 ± 0.15 vs. 0.68 ± 0.12 × 10^−3^ mm^2^/s; *p* = 0.1), D_fast_ (20.3 ± 6.6 vs. 24.9 ± 6.14 × 10^−3^ mm^2^/s; *p* = 0.2), and f_fast_ (0.41 ± 0.15 vs. 0.45 ± 0.12; *p* = 0.2) between patients with and without an event.

## Discussion

This is the first study to investigate the predictive value of FALD as defined by hepatic MRI-derived IVIM-modelled DWI parameters for the occurrence of adverse events during follow up in patients with a Fontan circulation. In our cohort of Fontan patients, we could not demonstrate an association between the DWI-derived degree of hepatic congestion and fibrosis and the occurrence of one of the following adverse events: death, listing for transplantation, or Fontan-related hospitalizations.

**Figure 2:**
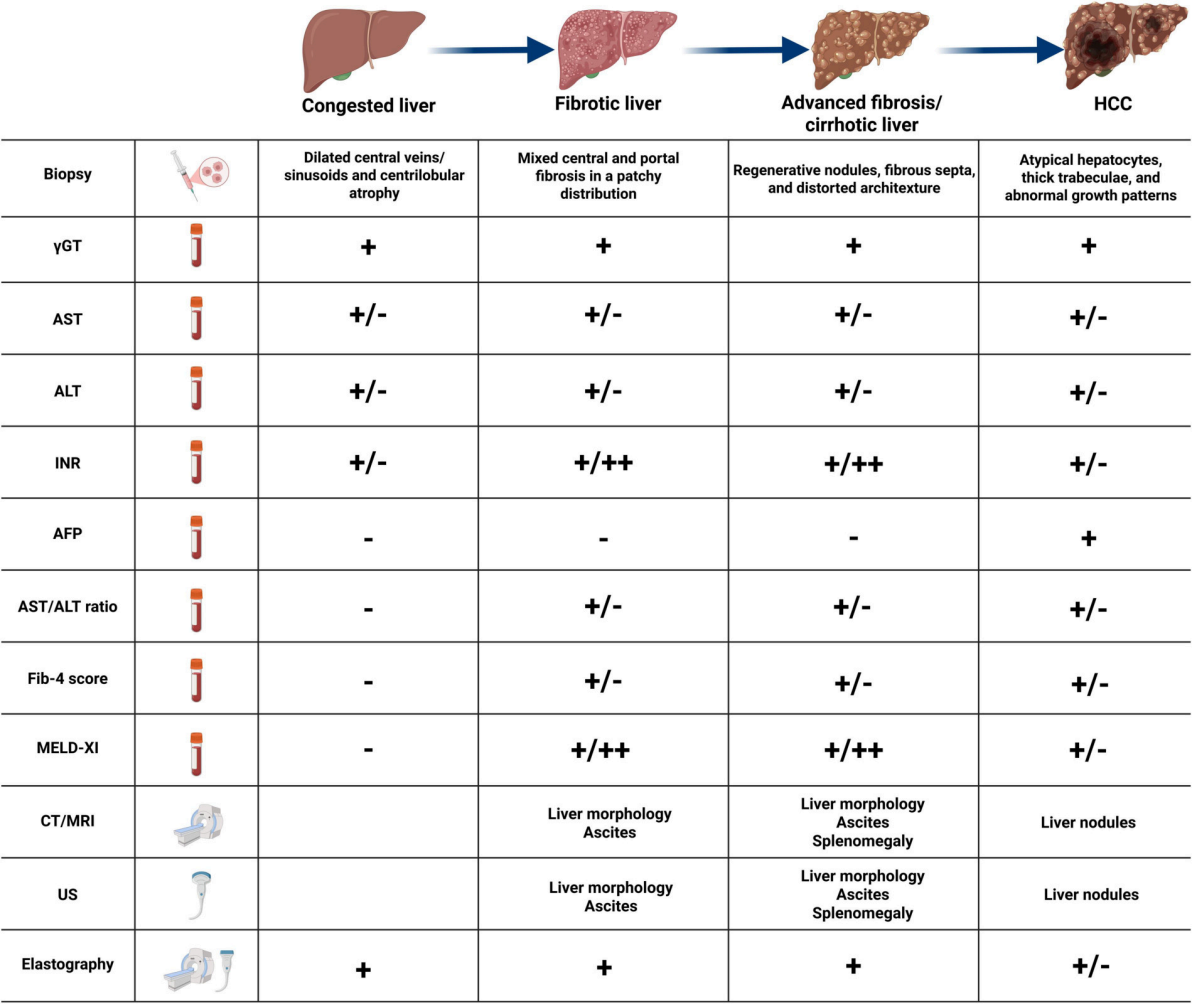
Limitations of the different diagnostic tools that are currently recommended in the assessment of FALD. The blue arrows show the natural development of FALD. Values with—indicate that the parameter is within normal limits and not elevated. Values with **+/-** suggest that the parameter may be mildly elevated. Values with **+** denote a define elevation, indicating abnormality. Values with **++** represent a marked or significant elevation. *γGT*, gamma-glutamyl transferase; *AST*, aspartate transaminase; *ALT*, alanine transaminase; *γGT*, gamma-glutamyl transferase; *INR*, internationalized-normalized ratio; *AFP*, alpha-fetoprotein; *MELD-XI*, model for end-stage liver disease excluding internationalized-normalized-ratio; *CT*, computer-tomography; *MRI*, magnetic resonance imaging; *US*, ultrasound.

FALD monitoring is recommended as part of the general follow-up of patients with a Fontan circulation (10). The goal is to detect advanced fibrosis early to allow for interventions that can optimize the Fontan circulation, thereby preventing or slowing progression to advanced cirrhosis, HCC, and other adverse events (3, 6). However, most non-invasive monitoring tools are unable to distinguish hepatic congestion, a condition inextricably linked to the Fontan circulation, from fibrosis/cirrhosis (Figure 2).

By allowing for non-invasive differentiation between hepatic congestion and fibrosis, IVIM-modelled DWI addresses the key diagnostic challenge in FALD. Standard ADC measurements on DWI reflect both molecular diffusion and microperfusion. Intravoxel incoherent motion model separates these components into distinct parameters: D_slow_ reflects true molecular diffusion and is associated with cellular changes and fibrogenesis; D_fast_ represents capillary blood flow velocity and thus microperfusion; and F_fast_ indicates the proportion of signal arising from microperfusion (17–19). Previously, we demonstrated that decreased ADC values primarily reflect reduced hepatic microperfusion (i.e., hepatic congestion), evidenced by a 38% reduction in D_fast_ and 33% reduction in F_fast_, rather than changes in molecular diffusion (13). Importantly, D_slow_ was initially comparable between Fontan patients and controls. However, with increasing time since Fontan completion, both ADC and D_slow_ progressively declined, while D_fast_ and D_fast_ remained stable. Together, these persistent microperfusion deficits and the progressive decline of molecular diffusion suggest that early congestion-related changes in blood flow are followed by intracellular remodelling consistent with fibrogenesis (13, 17). In contrast to other non-invasive diagnostic modalities, which are limited in their ability to identify tissue fibrosis due to the confounding effect of venous congestion, we hypothesised that IVIM modelled DWI would allow better prediction of adverse effects during follow up. Nonetheless, the current study could not associate either D_fast_ or D_slow_ with adverse events during a median follow-up of 9 years. In other words, we did not find an association of either hepatic venous congestion or fibrosis with outcomes.

Current guidelines recommend frequent and intensive FALD surveillance (6, 7). The goals of such surveillance would be early detection of liver disease that allows interventions to prevent adverse outcomes. However, this only holds when the detected liver abnormalities predict such outcomes. Another goal of FALD surveillance is to identify patients who may benefit from early referral for heart or combined heart-liver transplantation. Yet, a recent study found that regression of biopsy-proven liver fibrosis might be possible after successful heart transplantation (20). The results in the current study underscore the scarcity of evidence linking FALD monitoring—using current non-invasive modalities—with progression to advanced cirrhosis, HCC, or other adverse clinical outcomes. In FALD, standard biomarkers of hepatic injury correlate poorly with fibrosis, with often normal AST and ALT levels and elevated *γ*GT which lacks association with fibrosis or cardiac function (10). Hyperbilirubinemia, hypoalbuminemia, and prolonged INR are common but multifactorial, limiting their diagnostic value (10, 21). Additionally, composite scores have limited utility in FALD; only MELD-XI shows a modest association with fibrosis, without validated thresholds, while the aspartate aminotransferase-to-platelet ratio (APRI) and the Fibrosis-4 (FIB-4) index demonstrate limited discriminative performance for advanced fibrosis (11, 12, 22, 23). Moreover, imaging and transient elastography (TE) (Fibroscan) are confounded by congestion (3, 10, 24–26). Interestingly, even the severity of histologically assessed hepatic fibrosis—currently the gold standard for the detection of advanced fibrosis and cirrhosis in FALD—does not predict clinical outcome (27). Only alpha fetoprotein (AFP), a tumour biomarker, remains relevant for FALD surveillance, where levels ≥10 ng/ml have been associated with a 26-fold increased risk for developing HCC (28). This suggests that Fontan outcomes are mainly driven by the circulatory and haemodynamic alterations inherent to the Fontan circulation.

In light of the current study, the absence of a consistent relationship between FALD severity and prognosis in Fontan patients highlights two main paths for the course of FALD monitoring. First, if existing diagnostic methods are not associated with outcome, efforts should be directed to developing non-invasive tools to improve assessment of hepatic fibrosis and cirrhosis in congested livers and better predict patient outcomes. Second, the current recommended FALD surveillance strategy imposes a substantial burden on both patients and the current healthcare system. Individuals with a Fontan circulation already require intensive, multidisciplinary, lifelong follow-up, and the addition of additional hepatology visits and imaging further increases this burden. For many patients, particularly those with stable disease, these additional annual visits may be physically, emotionally, and logistically demanding, raising questions about the proportionality and efficiency of our current routine hepatic surveillance method. Therefore, reconsideration or re-evaluation of current recommendations for annual FALD surveillance is warranted.

### Strengths and limitations

Our study has strengths and limitations. This is the first study to investigate the prognostic value of hepatic DWI assessments in patients with a Fontan circulation. Of note, the studied cohort was relatively small. However, with the number of study participants and events in the primary endpoint we would have been able to detect an HR of approximately 0.34. The absence of a statistically significant association in our analyses therefore suggests that any potential true effect is likely to be small and of limited clinical relevance. However, as the measurements were performed using MRI techniques, we were unable to include any patients with a pacemaker. Additionally, we did not have liver biopsies to correlate our DWI measurements with the gold standard for the degree of hepatic fibrosis. Lastly, IVIM parameters can be sensitive to the specific MRI hardware and sequence settings, which might limit multi-centre generalizability. The advantage of the cohort used however, is the fact that it is a cohort of consecutive patients, with no sampling bias.

## Conclusion

IVIM-modelled DWI addresses a key diagnostic challenge in FALD by enabling distinction between hepatic congestion and fibrosis. However, even after this distinction, we did not find a relationship between IVIM-modelled DWI and clinical outcomes. These findings, in combination with current literature data, which also fail to demonstrate a relation between other non-invasive FALD assessment tools and outcome, raise the question whether the recommended intensive FALD monitoring strategies are justified.

## Data Availability

The raw data supporting the conclusions of this article will be made available by the authors, upon request and without undue reservation. Requests to access these datasets should be directed to Gaston van Hassel, g.van.hassel@umcg.nl.

## References

[1] Gewillig M Brown SC. The fontan circulation after 45 years: update in physiology. Heart. (2016) 102(14):1081–6. 10.1136/HEARTJNL-2015-307467 27220691 PMC4941188

[2] Rychik J Atz AM Celermajer DS Deal BJ Gatzoulis MA Gewillig MH. Evaluation and management of the child and adult with fontan circulation: a scientific statement from the American Heart Association. Circulation. (2019) 140(6):E234–84. 10.1161/CIR.0000000000000696 31256636

[3] Gordon-Walker TT Bove K Veldtman G. Fontan-associated liver disease: a review. J Cardiol. (2019) 74(3):223–32. 10.1016/j.jjcc.2019.02.016 30928109

[4] Keung CY Zentner D Gibson RN Phan D-KH Grigg LE Sood S. Fontan-associated liver disease: pathophysiology, investigations, predictors of severity and management. Eur J Gastroenterol Hepatol. (2020) 32(8):907–15. 10.1097/MEG.0000000000001641 31851099

[5] Goldberg DJ Surrey LF Glatz AC Dodds K O'Byrne ML Lin HC. Hepatic fibrosis is universal following fontan operation, and severity is associated with time from surgery: a liver biopsy and hemodynamic study. J Am Heart Assoc. (2017) 6(5):1–8. 10.1161/JAHA.116.004809 PMC5524062 28446492

[6] Baumgartner H de Backer J Babu-Narayan SV Budts W Chessa M Diller G-P. 2020 ESC guidelines for the management of adult congenital heart disease. Eur Heart J. (2021) 42(6):563–645. 10.1093/eurheartj/ehaa554 32860028

[7] Fuller S Kittleson MM Liaison JC Bradley EA Valente AM. 2025 ACC/AHA/HRS/ISACHD/SCAI guideline for the management of adults with congenital heart disease. J Am Coll Cardiol. (2026) 87(7):822–976. 10.1016/j.jacc.2025.09.006 41411480

[8] Emamaullee J Zaidi AN Schiano T Kahn J Valentino PL Hofer RE. Fontan-associated liver disease: screening, management, and transplant considerations. Circulation. (2020) 142(6):591–604. 10.1161/CIRCULATIONAHA.120.045597 32776846 PMC7422927

[9] Téllez L Rodríguez de Santiago E Minguez B Payance A Clemente A Baiges A. Prevalence, features and predictive factors of liver nodules in fontan surgery patients: the VALDIG fonliver prospective cohort. J Hepatol. (2020) 72(4):702–10. 10.1016/j.jhep.2019.10.027 31726116

[10] Téllez L Payancé A Tjwa E del Cerro MJ Idorn L Ovroutski S. EASL-ERN position paper on liver involvement in patients with fontan-type circulation. J Hepatol. (2023) 79(5):1270–301. 10.1016/j.jhep.2023.07.013 37863545

[11] Emamaullee J Khan S Weaver C Goldbeck C Yanni G Kohli R. Non-invasive biomarkers of fontan-associated liver disease. JHEP Reports. (2021) 3(6):100362. 10.1016/j.jhepr.2021.100362 34693238 PMC8517550

[12] Téllez L Rincón D Payancé A Jaillais A Lebray P Rodríguez de Santiago E. Non-invasive assessment of severe liver fibrosis in patients with fontan-associated liver disease: the VALDIG-EASL FONLIVER cohort. J Hepatol. (2025) 82(3):480–9. 10.1016/j.jhep.2024.09.005 39260705

[13] Dijkstra H Wolff D Van Melle JP Bartelds B Willems TP Oudkerk M. Diminished liver microperfusion in fontan patients: a biexponential DWI study. PLoS One. (2017) 12(3):1–13. 10.1371/journal.pone.0173149 PMC5336266 28257439

[14] Shiraga K Ono K Inuzuka R Asakai H Ookubo T Shirayama A. Intravoxel incoherent motion imaging has the possibility to detect liver abnormalities in young fontan patients with good hemodynamics. Cardiol Young. (2019) 29(7):898–903. 10.1017/S1047951119001070 31250776

[15] Lu CW Wu CH Huang MT Lee CS Chen HL Lin MT. Liver fibrosis detected by diffusion-weighted magnetic resonance imaging and its functional correlates in fontan patients. Eur J Cardio-thoracic Surg. (2024) 66(1), ezae249. 10.1093/ejcts/ezae249 38913856

[16] Wolff D van Melle JP Bartelds B Ridderbos FJS Eshuis G van Stratum EBHJ. Fontan circulation over time. Am J Cardiol. (2017) 120(3):461–6. 10.1016/J.AMJCARD.2017.05.005 28624095

[17] Wolff D Van Melle JP Dijkstra H Bartelds B Willems TP Hillege H. The fontan circulation and the liver: a magnetic resonance diffusion-weighted imaging study. Int J Cardiol. (2016) 202:595–600. 10.1016/j.ijcard.2015.09.088 26447669

[18] Ren H Xu H Yang D Tong X Zhao X Wang Q. Intravoxel incoherent motion assessment of liver fibrosis staging in MASLD. Abdom Radiol. (2024) 49(5):1411–8. 10.1007/s00261-024-04207-w 38461432

[19] Li YT Cercueil JP Yuan J Chen W Loffroy R Wáng YXJ. Liver intravoxel incoherent motion (IVIM) magnetic resonance imaging: a comprehensive review of published data on normal values and applications for fibrosis and tumor evaluation. Quant Imaging Med Surg. (2017) 7(1):59–78. 10.21037/qims.2017.02.03 28275560 PMC5337188

[20] Accord RE Cuperus FJC Hoendermis E Mariani M Mecozzi G Nijkamp MW. Initial experience with combined heart-liver transplantation in The Netherlands: exploring the boundaries of isolated and combined transplantation. Netherlands Hear J. (2025) 33(9):250–8. 10.1007/s12471-025-01969-w PMC12364779 40694181

[21] Surrey LF Russo P Rychik J Goldberg DJ Dodds K O'Byrne ML. Prevalence and characterization of fibrosis in surveillance liver biopsies of patients with fontan circulation. Hum Pathol. (2016) 57:106–15. 10.1016/j.humpath.2016.07.006 27476041

[22] Martin De Miguel I Kamath PS Egbe AC Jain CC Cetta F Connolly HM. Haemodynamic and prognostic associations of liver fibrosis scores in fontan-associated liver disease. Heart. (2023) 109(8):619–25. 10.1136/heartjnl-2022-321435 36581444

[23] Evans WN Acherman RJ Ciccolo ML Carrillo SA Galindo A Rothman A. MELD-XI Scores correlate with post-fontan hepatic biopsy fibrosis scores. Pediatr Cardiol. (2016) 37(7):1274–7. 10.1007/s00246-016-1428-1 27300556

[24] Sigrist RMS Liau J El Kaffas A Chammas MC Willmann JK. Ultrasound elastography: review of techniques and clinical applications. Theranostics. (2017) 7(5):1303–29. 10.7150/thno.18650 28435467 PMC5399595

[25] Egbe A Miranda WR Connolly HM Khan AR Al-Otaibi M Venkatesh SK. Temporal changes in liver stiffness after fontan operation: results of serial magnetic resonance elastography. Int J Cardiol. (2018) 258:299–304. 10.1016/j.ijcard.2018.01.108 29433966

[26] Munsterman ID Duijnhouwer AL Kendall TJ Bronkhorst CM Ronot M van Wettere M. The clinical spectrum of fontan-associated liver disease: results from a prospective multimodality screening cohort. Eur Heart J. (2019) 40(13):1057–68. 10.1093/eurheartj/ehy620 30346512

[27] Hilscher MB Wells ML Venkatesh SK Cetta F Kamath PS. Fontan-associated liver disease. Hepatology. (2022) 75(5):1300–21. 10.1002/hep.32406 35179797

[28] Ohuchi H Hayama Y Nakajima K Kurosaki K Shiraishi I Nakai M. Incidence, predictors, and mortality in patients with liver cancer after fontan operation. J Am Heart Assoc. (2021) 10(4):1–12. 10.1161/JAHA.120.016617 PMC7955326 33538186

